# Is Necessary Intraoprative Frozen Section in Sentinel Lymph Node Biopsy for Breast Cancer Patients?

**DOI:** 10.31557/APJCP.2020.21.3.647

**Published:** 2020-03

**Authors:** Gholamali Godazande, Siavash Moradi, Farshad Naghshvar, Leyla Shojaee

**Affiliations:** 1 *Department of Surgery, *; 2 *Department of Community Medicine, Gastrointestinal Cancer Research Center, *; 3 *Department of Pathology, Gut and Liver Research Center, Mazandaran University of Medical Science, Sari, Iran. *

**Keywords:** Breast cancer, sentinel lymph node biopsy, frozen section, false negative rate

## Abstract

**Background:**

Improvements in the process of staging and surgical treatment of axillary lymph nodes in recent years, have led to the use of intra operative frozen section pathology to examine the sentinel lymph node biopsy in breast cancer patients.

**Materials and Methods:**

we evaluated the results of the Sentinel biopsy in 102 patients with early stage breast cancer, which were negative clinical lymph nodes, and analyzing the true positive and false negative rate, diagnostic accuracy of frozen section lymph node biopsy. It also studied the factors affecting the sentinel and non-sentinel lymph nodes in patients treated by axillary lymph dissection.

**Results:**

In this study, we investigated 102 patients’ stage 1and 2 breast cancer with clinical negative axillary lymph node and candidates for sentinel lymph node biopsy, were placed under investigation. 15.7 % of the real positive results of sentinel and 62.7 % of the real negative and 2 % false positives and 20.9 % false negative results and% 78. 4 diagnostic accuracy, has been frozen section. Among the patients who were initially or delayed in the axillary dissection, 37% had more than two lymph nodes. While in general, 16.7% of patients had a need for axillary lymph node dissection based on z11 criteria. Lymph-vascular invasion was a major contributor to lentil involvement in Sentinel and non-Sentinel nodes.

**Conclusion:**

Frozen section pathology during the operation of sentinel lymph node biopsy has been initiated to prevent the need for a reoperation in early stage breast cancer patients. However, due to low tumor burden in patients who are candidates for this procedure, and the constraints in the initial sections and their false negative results, also the removal of frozen section will not have an effect on the rate of increasing reoperation and can be effective in reducing the time and cost of surgery.

## Introduction

The Sentinel Lymph Node Biopsy technique was performed by GIULIANO in evaluating the axillary lymph nodes and is currently being used as a standard method for staging the status of the axillary lymph nodes, replacing the axillary dissection in patients with early stage breast cancer and negative clinical lymph nodes(Giuliano et al., 1994; Lyman et al., 2005). Numerous large-scale studies have been performed in randomized trials, and it has been proved that in this group of breast cancer patients, in the case of negative lymph node. sentinel lymph node dissection is safe, and it is associated with the same results in disease free survival and overall survival, with less complications in the upper limbs (Krag et al., 2010; Giuliano et al., 2016). In the evaluation of Sentinel lymph node, Intraoperative frozen section is one of the standard methods for evaluating SLN. Up to 35% of the Sentinel lymph nodes require axillary dissection(Veronesi et al., 2003). The findings of the trial Z11 indicated that early stage breast cancers, who are candidates for breast conserving surgery and radiotherapy and have a low tumor burden in the axillary lymph node (less than 2 metastatic lymph nodes), can be excluded from the axillary of dissection, and the results from the point of view locoregional relapse and survival were the same in the group without axillary dissection and patients with axillary dissection 

Also, IBCSG01-23 and AMAROS trials showed that patients with T1-2N0 treated with sentinel biopsy had a metastatic axillary lymph node, the results of survival and relapse in patients who received radiotherapy alone were identical to those that were axillary dissection but there were more less complications in the group receiving only radiotherapy. According to the results of these studies, in a study conducted by Julie et al, found that despite the effectiveness of use of intraoperative frozen section in sentinel lymph node biopsy, it tends to be used after the introduction of z11 trial, in early stage breast cancer patients who are candidates for breast conserving surgery and radiotherapy are reduced (Giuliano et al., 2011; Jorns and Kidwell, 2016). Augusto et al studied the role of frozen section in breast cancer patients. Based on this study, frozen had a low sensitivity to micro metastasis discovery (19%) but had a high sensitivity to macro metastasis discovery (75%). The majority of the false negative cases in the lymph nodes are smaller than 2.1 mm, and most patients were lobular carcinoma (Galimberti et al., 2013). According to the results of recent studies and trials in the treatment of axillary lymph node, the need for use of intraoperative frozen section in sentinel lymph node biopsy, which imposes costs and financial burden on the patient, has been questioned. In this study, the necessity of using intraoperative frozen section in sentinel lymph node biopsy in early stage breast cancer patients, which are negative clinical lymph nodes, is examined whether its effect on treatment decision is significant or gradually decreases. 

## Materials and Methods

In this study, 102 patients with early stage breast cancer, who had negative clinical lymph nodes, were examined and treated at Imam Khomeini Hospital in Sari between 2016-2018. All patients were candidates for breast conserving surgery and Sentinel lymph node biopsy. This study was approved by the Cancer Research Center of Mazandaran University of Medical Science. Demographic and pathological data of the patients were recorded (Table 1). Patients with DCIS or receiving chemotherapy were excluded. Also, patients who did not detect lymph node Sentinel during the procedure and were exposed to axillary dissection were excluded. All patients were subjected to a radioisotope injection (Tc 99) a few hours before the operation by a nuclear medicine specialist in the peri areole or the peritumoral region of the breast. After lymphocyteography and the detection of lymph node Sentinel, the patient was transferred to the operating room. Prior to the surgery, first with the help of the gamma scanner, lymph node Sentinel was detected. Lymph nodes that had more than 10% maximum radiation absorption at the injection site were sent to the Department of Pathology for evaluation of the frozen pathology. In the pathology section, in lymph nodes smaller than 1 cm, with one cut and lymph nodes larger than two centimeters, was done with two cuts. Half of the lymph nodes were under the study of frozen pathology. It was freezing in the negative temperature of 20°C and then underneath H and E stain and was observed by an experienced pathologist using a microscope. After initial evaluation, to determine the definitive pathology, specimen was fixed in formalin, the remaining half of the lymph node was fixed to determine the definitive pathology in formalin. After the final pathology report in the case of metastatic involvement, delayed 2 weeks after the first surgery, they underwent an axillary re-operation and became lymph node dissection. The final results of the study of the number of sentinel and non-sentinel axillary lymph nodes in patients who were initially or delayed in the detection of lymph nodes were investigated. Factors influencing the probability of sentinel and non-sentinel lymph nodes lymph node involvement were analyzed. Descriptive qualitative variables were studied with frequency (percent). Also, the frequency difference between the two groups of SLN and ALND was measured by chi-squared statistical test and double-sided p-value less than 0.05 was considered statistically significant. The online software (= https: // ebm / tools. Knowledge translation. Net / calculator / diagnostic) was used to examine the diagnostic aspects of Frozen Sections.

## Results

102 patients between 24-79 years with an average age of 49 years with early stage breast cancer, who were negative clinical lymph nodes and under the sentinel node biopsy were studied. 97% histopathology was invasive ductal carcinoma. 26.5% were less than 40 years old and 73.5% were over 40 years old. All patients underwent breast conserving surgery and after radioisotope injection (technetium 99) intraoperative sentinel lymph node biopsy and frozen section examination. In 15/7 % of the patients, the frozen section pathology results were positive, which was followed by axillary lymph node dissection. In 64 (62.7%) of patients, negative lymph nodes were reported(true negative), and intraoperative dissection was not performed, and 21( 20.6%) had a false negative result, 1(0.98%) had a false positive and 16(15.7%) true positive. All of patients who reported false negatives in frozen had been delayed by axillary dissection. Finally, 37.3% of this patient underwent axillary dissection, and 62.7% of patients did not receive axillary dissection. Of the 16 patients who were initially frozen positive and were subjected to dissection, 10 patients had more than 3 lymph nodes, and 6 patients were less than 3 and of 21 patients with false negative frozen and delayed axillary dissection Only 4 patients had more than two lymph nodes and the rest of the patients were less than or equal to two lymph nodes. 

Only 14 patients (13.7 %) needed axillary dissection if it was not used with frozen and the final decision was taken later and after the pathologic report, according to the pattern reported in the z11 study, less than two lymph nodes were diagnosed in patients with less than two lymph nodes, only 14 (13.7%) patients need to have an axillary dissection. In general, 580 lymph nodes were removed, 84 of which were positive and 496 were negative.

The diagnostic accuracy of frozen section was 78.4%. In evaluating the factors affecting lymph node involvement in sentinel and non-sentinel axillary lymph nodes, including the tumor size, patient’s age, the type of pathology of the tumor and Lymph-vascular invasion, in the evaluation of this study, Lymph-vascular invasion has been a contributing factor in predicting the involvement of non-sentinel axillary lymph nodes (Table2) 

**Table 1:** Frequency of Clinical and Pathologic Features of Patients

Clinicopathologic characteristics	Frequency (present)
Age group	
Less than 40y.	27 (26.5)
40y. and more	75 (73.5)
Tumor size	
Less than 2 cm^3^	22 (21.6)
2 cm^3^ and more	80 (78.4)
Nodal status	
Positive	49 (48.0)
Negative	53 (52.0)
Historicaltype	
IDC	91 (89.2)
MC	3 (2.9)
DCIS	4 (3.9)
TC	1 (1.0)
ILC	3 (2.9)
Lympho-vascular invasion	
Positive	47 (46.1)
Negative	55 (53.9)
Axillary lymgh node dissection	
Yes	38 (37.3)
No	64 (62.7)

**Figure 1:**
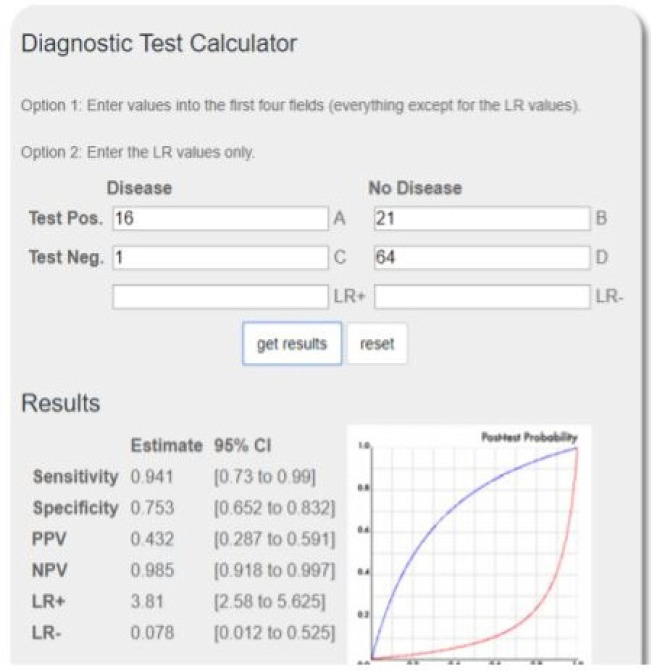
Diagnostic Accuracy of Frozen Section

**Table 2:** Investigating the Factors Influencing Lymph Involvement of Sentinel and Non-Sentinel Axillary

Clinicopathologic characteristics	Frequency (percent)					
	SLN		P-Value*	ALND		P-Value*
	Yes	No		Yes	No	
Age group						
Less than 40 y.	10 (27)	17 (26.2)	0.928	14 (29.8)	13 (23.6)	0.508
40y. and more	27 (73)	48 (73.8)		33 (70.2)	42 (76.4)	
Tumor size						
Less than 2 cm^3^	8 (21.6)	14 (21.5)	0.992	13 (27.7)	9 (16.4)	0.228
2 cm^3^ and more	29 (78.4)	51 (78.5)		34 (72.3)	46 (83.6)	
Histological Type						
IDC	33 (89.2)	58 (89.2)	0.087	42 (89.4)	49 (89.1)	0.628
MC	1 (2.7)	2 (3.1)		1 (2.1)	2 (3.6)	
DCIS	0 (0)	4 (6.2)		3 (6.4)	1 (1.8)	
TC	0 (0)	1 (1.5)		0 (0)	1 (1.8)	
ILC	3 (8.1)	0 (0)		1 (2.1)	2 (3.6)	
Lympho-vascular invasion						
Positove	14 (37.8)	24 (36.9)	0.927	32 (68.1)	6 (10.9)	Less than 0.001
Negative	23 (62.2)	41 (63.1)		15 (31.9)	49 (89.1)	

## Discussion

The reason for the use of intraoperative frozen section is that axillary sentinel lymph node is considered in preventing re-operation in early breast cancer patients. But has low sensitivity in the evaluation of lymph nodes with low tumor burden (micro metastasis less than 2 mm). One of the causes of this, is the limitation in the cutting of lymph nodes and the possibility of false negative, and ultimately leads to re-surgery (Liu et al., 2011). Most studies reported the real negative rate of frozen about 25 %. The false negative value of frozen reported between 10 and 60 %, but most studies reported it between 15 - 20 % (Holck et al., 2004; Geertsema et al., 2010; Jensen et al., 2010; Chan et al., 2011; Weber et al., 2012; Poling et al., 2014; Wong et al., 2015). In a meta-analysis, the sensitivity of intraoperative frozen section was reported in 62%-76% of lymph node evaluations (Chao et al., 2001; Holck et al., 2004; Wada et al., 2004). In other studies, the rate of false negative frozen was 5-43% (Tew et al., 2005).

In a study conducted in August 2018 by Augusto Lombardi et al., (2018) Frozen section was a lower sensitivity to micro metastasis assessment (19%) and has a higher ability to detect macro-metastasis (75%). The study also concluded that if the results of the IBCSG 23-10 assay were used and the axillary dissection was performed in patients with macro-metastasis, the rate of axillary surgery would decrease from 12% to 4 %.

In this study, we evaluated the false negative rate of frozen section pathology in patients who were exposed to sentinel lymph node biopsy. The false negative value is estimated at 20 %. The reason for this can be the limitation of the number of lymph nodes in the evaluation of frozen and low tumor burden in lymph node sentinel. In general, low frozen sensitivity is attributed to several factors in a number of studies. Like the Misinterpretation sampling method, especially the use of IHC in the definitive study of lymph node sentinel, using this method, 25% of patients with sentinel metastasis included isolated tumor cells, but in studies where the final evaluation was not based on IHC, the frozen sensitivity has been high in the discovery of lymph node metastasis. In addition, tumor size and OMS are related variables. The type of lobular pathology has also been effective in detecting sentinel metastasis intraoperative in the frozen sensitivity (Gawlick et al., 2008; Moatasim et al., 2013). In our study, 15/71% (16 patients) had a positive lymph node sentinel in the evaluation of frozen, which were subjected to dissection. In the final evaluation of the pathology in this patients, 10 patients had more than 3 lymph node involvement, and 6 patients had less than 3 nodes positive. In patients who reported lymph node involvement after the final report of the pathology of lymph node sentinel, 17 were less than 3 lymph nodes involvement and 4 patients had more than 3 lymph nodes involvement. Finally, if after evaluating Sentinel lymph node biopsy, the decision for axillary dissection was delegated after a definitive pathology report; and the Z11 criteria, (dissection; if more than 2 metastatic lymph nodes) were considered, only 13.7% of our patients needed axillary dissection while 37.3% of the patients were lymph node dissection. In fact, if after SLN, surgical lymph node dissection decision was postponed to a later pathologic, in terms of reduction of axillary dissection, costly and duration and were prevented in axillary dissection, about 23.6% of patients based on Z11 criteria. The diagnostic accuracy of frozen in this study was 78/4%. In evaluating the factors influencing the sentinel and non-sentinel axillary lymph node involvement, Lymph-vascular invasion was the only variable that had a significant effect and variables such as age, tumor size, and type of pathology had no significant relationship with the sentinel and non-sentinel lymph node involvement. Although our study showed that the frozen section lymph node sentinel axillary has a sensitivity of 95% and specificity 75%, however, according to numerous studies and trials of z11 and Amaros, the effect of the role of radiotherapy as an alternative method of axillary dissection in patients with limited tumor burden in axillary and without changing the risk of relapse of the disease by altering disease free survival and overall survival, in the case of removal of the use of frozen section for the study of SLN, not only does not increase the need for re-surgical procedures, but the use of frozen may actually lead to unnecessary axillary surgery and impose more financial burden on the patient and the system of healing and increased time of surgical procedures. It can be said that patients with early stage breast cancer, which axillary lymph nodes are negative in clinical examination, and no suspicious lymph nodes have been reported in the imaging study it can be indicative of a lack of involvement tumor or metastasis lymph node with low tumor burden. Therefore, either the sentinel lymph node is a real negative or false negative. In both cases, axillary surgery is not initially performed, maybe there is a positive real possibility with limited involvement and micro metastasis or involvement may be less than two lymph nodes which can be discarded from axillary dissection and the patient is under radiotherapy. False negative patients that are more likely to detect metastasis like micro metastasis or isolated tumor cell or have less 2 lymph nodes involvement, however, re-surgical procedures can be discarded. In fact, the use of new protocols to deal with different situations of lymph node involvement can prevent the complications of axillary dissection and reduce the stress of patients due to the need for re-surgery, and therefore the use of frozen section is not necessary and in this study, has not reduced the risk of re-surgery (Russo et al., 2017; van der Noordaa et al., 2017). One of the shortcomings of this study was the low number of patients. Can be found in a study with larger sample size and more accurate pathologic examination of metastatic lymph nodes and perform IHC, also use Z11 criteria, to achieve more accurate results.

A sentinel Lymph node biopsy is a reliable method for evaluating the status of axillary lymph nodes, and is an appropriate alternative to excisional staging, and reduction of the complications of axillary dissection. With regard to the results of new trials in the treatment of lymph node involvement with low tumor burden, the use of frozen section pathology has been questioned more than ever. In this study, due to the false negative frozen and the lack of value of this method in reducing the risk of re-surgery, and in some cases, the decision to axillary dissection with the consideration of the positive of the sentinel, regardless of the number of involved node, can be recommended in patients early stage breast cancer with negative clinical lymph nodes , are more appropriate, it is not decided to axillary dissection by frozen, and after Definite pathology of sentinel lymph node and considering the criteria of z11, dissection is done.
